# Endophyte-Mediated Green Synthesis of Metallic Nanoparticles: Mechanistic Insights, Antibiofilm Efficacy, and Translational Challenges

**DOI:** 10.3390/ph19101515

**Published:** 2026-09-23

**Authors:** Kaniska Singh, Ayush Kashyap, Mohammad Arham Siddiqui, Shabaaz Begum Jameel Pasha, Prateek Gururani, Avinash Sharma, Mohammad Azam Ansari, Hajed Obaid A. Alharbi, Arshad Husain Rahmani

**Affiliations:** 1 Department of Microbiology, Graphic Era (Deemed to be University), Dehradun 248002, India; 2 Department of Biotechnology, Graphic Era (Deemed to be University), Dehradun 248002, India; 3 Department of Epidemic Disease Research, Institutes for Research and Medical Consultations (IRMC), Imam Abdulrahman Bin Faisal University, Dammam 31441, Saudi Arabia; maansari@iau.edu.sa; 4 Department of Medical Laboratories, College of Applied Medical Sciences, Qassim University, Buraydah 51452, Saudi Arabia

**Keywords:** antimicrobial resistance, biofilm, endophyte, metallic nanoparticles, reactive oxygen species

## Abstract

Antimicrobial resistance, a major health problem for the world’s population, is caused by pathogenic bacteria capable of forming biofilms, claiming 4.95 million lives each year, according to WHO (World Health Organization) data, and could escalate up to 10 million annual deaths by 2050 if unchecked. Biofilms are the structured microbial populations surrounded and composed by an EPS (Extracellular Polymeric Substance) matrix to protect themselves from host immune responses and antibiotics, which makes the infections persistent and difficult to treat. Current global approaches to the pathogenic microbes to address AMR (Antimicrobial Resistance) are solved by different alternative strategies; one such strategy is nanotechnology, a rapidly developing field in the twenty-first century, using metallic nanoparticles that are generally characterized by dimensions within the range of 1–100 nm, have distinct structural and functional properties from bulk materials, and perform higher penetration in the cell membranes of pathogenic microbes. Overall, the present review offers a detailed overview of significant knowledge gaps regarding scalability, long-term safety, and the mechanistic pathway of nanoparticles synthesized from endophytic microorganisms. The inclusion also summarizes a comparison of bacterial and fungal endophyte-mediated metallic nanoparticles with plant-mediated metallic nanoparticles, particularly in terms of metabolite diversity, synthesis efficiency, size, nanoparticle stability, toxicity, antimicrobial activity, and antibiofilm potential. The novelty lies in how the efficacy of endophytic-mediated metallic nanoparticles acts against the defense mechanisms of pathogenic microorganisms in terms of molecular pathways and processes associated with ROS (Reactive Oxygen Species), disruption of membrane integrity, quorum sensing interference, EPS (Extracellular Polymeric Substance) degradation, DNA and protein disruption, and biofilm collapse. However, nanoparticles synthesized from endophytic microorganisms face translational challenges and limitations that need to be addressed.

## 1. Introduction

The rapid increase in the human population worldwide has intensified several challenges, including major public health concerns, AMR (Antimicrobial Resistance), and associated infections in agriculture and the broader environment [1,2]. AMR is now identified as one of the major public health challenges of the twenty-first century, largely influenced by the inappropriate and excessive use of antibiotics [3]. Microbial resistance represents a natural phenomenon in microorganisms, and its spread has accelerated drastically over the past three decades as a result of the overuse and misuse of antibiotics in plants, animals, and humans [4]. In response, evidence-based antibiotic prescribing guidelines that are regularly updated are essential for improving antibiotic use and limiting resistance [5]. However, even higher antibiotic doses often fail to eliminate pathogens because most of the microbes can protect themselves through quorum sensing, biofilm development, and the production of virulence determinants that help them survive antimicrobial stress. For example, *Pseudomonas aeruginosa*, a common cause of respiratory infections, is well known for its ability to withstand antibiotic treatment and persist in hostile environments [6]. Such survival strategies are also seen in ESKAPE (*Enterococcus faecium*, *Staphylococcus aureus*, *Klebsiella pneumoniae*, *Acinetobacter baumannii*, *Pseudomonas aeruginosa*, and *Enterobacter species*), pathogens that can be responsible for persistence and severe infections [7]. Natural bioactive compounds such as mangiferin have also been reported to inhibit quorum sensing-regulated biofilm formation, providing an example of how bioactive molecules can interfere with bacterial signaling [8]. The development of biofilm-forming AMR pathogens is associated with extracellular matrix production, altered microenvironments, quorum sensing, genetic adaptation, and efflux pump-mediated tolerance [9]. Taken together, AMR represents a serious global threat that requires innovative solutions beyond conventional antibiotics [10,11].

Endophytes are an effective source of biologically active metabolites, acting as a natural form of inhibitors that can be impactful in addressing antibiotic-resistant pathogens of plants and humans [12]. Endophytes are symbiotic microbes that inhabit plant tissues intracellularly or intercellularly without harming the host [13]. They improve plant growth by secreting plant hormones like gibberellins, auxins, and cytokinins [14]. In agriculture, endophytic microbes perform a pivotal function in plants by their rapid proliferation and environmental adaptability [15]. Endophytic species such as *Bacillus* and *Pseudomonas* produce metabolites that may exhibit biofilm inhibition [16]. Primarily addressing unique features of extracellular and intracellular enzymes of endophytic bacterial and fungal species with nanotechnology, these endophytes synthesize numerous metallic nanoparticles [17,18]. Traditional benefits of the medicinal values of plants discovered in India are known for their ethnobotanical properties that leverage the rich diversity of bioactive phytochemicals associated with endophytic microorganisms [19]. Recent research has shifted towards the endophytic microorganisms associated with medicinal plants that possess the ability to biosynthesize metallic nanoparticles, highlighting the benefits of endophytic-mediated metallic nanoparticles. For example, AgNPs (Silver Nanoparticles) were synthesized from the endophytic fungus *Cornespora smithii* isolated from *Bergenia ciliate* [20].

The field of nanotechnology emerges as a promising alternative, providing cutting-edge strategies to address resistant pathogens [21]. Over the last decade, metallic nanoparticles have been used for their distinctive nanoscale physical and chemical properties, which have gained attention for preventing, treating, and diagnosing infectious diseases [22]. Usually, common metallic nanoparticles (silver, gold, zinc, iron, titanium, calcium, and palladium) are synthesized via physical, chemical, and biological routes [23]. The biological synthesis approach is favored for its low toxicity, energy efficiency, eco-friendliness, and cost-effectiveness [24]. Studies have shown that nanoparticles produced by endophytic microorganisms can provide a range of useful biological effects, such as activity against multidrug-resistant pathogens, anticancer potential, and improved plant growth and protection through better nutrient uptake and resistance to pathogens. Nanoparticles, including metals, metal oxides, liposomes, and nano-polymers, have indicated antibiofilm and antimicrobial activities [25]. Nano based therapeutics studies were also conducted to address environmental antimicrobial resistance [26]. The synthesized nanoparticles were also used for the purification of drinking water by combating waterborne pathogens [27]. Some of the metallic nanoparticles were analyzed for biofilm inhibition, which caused antibiotic resistance against all the strains of *Escherichia coli* [28]. Beyond infection control, these materials aid in heavy metal removal, pollutant degradation, and water purification, though their environmental stability and persistence, transport, and the long-term effects on ecosystems are important for considerations [29,30].

Most of the existing reviews on green-synthesized nanoparticles focus broadly on plant extracts, microbial synthesis, or general antimicrobial activity [31,32,33]. This review is uniquely positioned because it integrates four interconnected aspects that are often discussed independently such as the mechanistic and symbiotic roles of endophytes together with their ecological and biological significance; the antibiofilm activity of endophytes against pathogenic microorganisms; advances over the past decade in endophyte-mediated metallic nanoparticle syntheses and their role in inhibiting biofilms formed by pathogenic microorganisms; and a comparative evaluation of endophyte-mediated metallic nanoparticles and plant-mediated metallic nanoparticles. According to the recent descriptive studies that were reported, the novelty in the present review is an in-depth study of the antibiofilm activity of endophyte-mediated synthesis of metallic nanoparticles. This shifts the entire manuscript towards the central novelty from a descriptive review of metallic nanoparticle syntheses to a critical review of how endophyte-derived metallic nanoparticles may interfere with microbial survival strategies, with their challenges and future directions.

The relevant studies were identified through database searches, including ACS Nano, Nano Today, Web of Science, Scopus, PubMed/MEDLINE, and Google Scholar, covering publications from 2016 to 2026, searched during the period between 1 January 2026 and 15 July 2026. Search terms included different combinations of “green synthesis,” “biosynthesis,” “phytofabrication,” “endophytic fungi,” “metal nanoparticles,” “zinc oxide nanoparticles,” “silver nanoparticles,” “antimicrobial,” “antibiofilm,” and “biomedical applications,” combined using operators. Studies were included if they reported original experimental data or comprehensive reviews on plant- or microorganism-mediated nanoparticle synthesis and their biomedical evaluation.

## 2. Endophytic Microorganisms: The Symbiotic Role, Functional Mechanism, and Ecological Significance

Endophytic bacteria and fungi reside within healthy plant tissues without adversely affecting the plant [34]. In a symbiotic sense, the plant provides these microorganisms with nutrients, shelter, and a chemically rich internal habitat, while the endophytes return benefits through nutrient mobilization, growth promotion, metabolic support, and stress tolerance for better crop production [35]. Endophytes act as a source of bioactive compounds that have shown promising results in reducing the dependence of agrochemicals and advancing sustainable agriculture [36]. For example, *Pseudomonas granadensis* CT364, an endophyte isolated from olive trees, primarily colonizes the rhizosphere. Genomic and functional analyses have demonstrated its plant growth-promoting characteristics [37].

Additionally, *Bacillus cereus* BI-8 and *Bacillus subtilis* BI-10 endophytes promote plant growth by producing IAA (Indole-3-Acetic Acid) and ammonia, and showed maximum productivity in enzymatic activities [38]. A study on *Arachis hypogaea* L. (peanut) demonstrated that salt-tolerant endophytes such as *Bacillus tequilensis*, *Staphylococcus epidermidis*, and *Bacillus siamensis* enhanced germination, plant height, biomass, antioxidant enzyme activity, root surface area, and defense-related gene expression under salt stress, showing how endophytes can translate microbial traits into whole-plant resilience [39]. Endophytic microorganisms are increasingly recognized not only as intimate symbionts or plant growth regulators but also as promising sources of antibiofilm and antimicrobial resistance interventions [40]. Recent studies have shown that endophytic bacteria and fungi associated with medicinal and native plants produce secondary metabolites that strongly inhibit MDR (Multidrug-Resistant) pathogens and their biofilms [16]. For example, endophytic bacteria from *Archidendron pauciflorum* yielded extracts with low MIC (Minimum Inhibitory Concentration) and MBC (Minimum Bactericidal Concentration) values against MDR (Multidrug Resistance) strains and reduced biofilm formation by 52% and eradicated all established biofilms by more than 42% [41]. Furthermore, endophytes act as hidden chemical entities whose secondary metabolites can target multiple biofilm-associated microbes, including exopolysaccharide synthesis, efflux pumps, and oxidative stress responses, thereby sensitizing biofilm-embedded cells [42]. Despite this potential, translating endophyte-based antibiofilm strategies into clinical or industrial products faces several challenges, such as variable colonization efficacy, difficulty in storage, strain-to-strain variability, and biosafety concerns [43]. Endophytic-mediated metallic nanoparticles offer potential advantages over direct use of endophytes for biofilm inhibition, primarily due to their nanoscale dimensions and multiple mechanisms generated at once, including broader membrane damage, enabling deep penetration into the protective EPS (Extracellular Polymeric Substance) matrix, and ROS (Reactive Oxygen Species) [25,44]. However, despite their potential, the practical use of endophytes as living agents remains constrained by several intrinsic limitations. These include variable colonization efficiency, host specificity, instability under field conditions, inconsistent metabolite expression, sensitivity to environmental fluctuations, difficulties in formulation and storage, and limited control over dose, delivery, and persistence within the host. Recent studies also showed that biosurfactant-capped silver nanoparticles synthesized using endophytic bacteria can inhibit *Staphylococcus aureus* and *Pseudomonas aeruginosa* and reduce biofilm biomass by ~40–60%, either comparable to or exceeding some conventional antibiotics [45]. These studies also highlight that endophyte-derived CuO, Au, Ag, ZnO, and Se nanoparticles display broad antibiofilm, antimicrobial, and even anticancer activities, with the added advantages of green synthesis [46,47]. Harnessing the direct application of endophyte-mediated metallic nanoparticles ending the critical gap in inhibition and control of biofilm-forming pathogens by concentrating bioactive metabolites to a most stable inorganic core, allowing precise size and surface specific penetration deep into persistent biofilms, triggering multiple synergistic attacks such as metal ion release, quorum sensing inhibition, ROS bursts, membrane rupture, and offering the most scalable and reliable production than treating with live endophytes [48,49,50]. Despite their promising properties, there are several challenges that remain to be solved before the widespread use of endophyte-mediated metallic nanoparticles, including the need to clarify the interaction of nanoparticles synthesized from endophytic microorganisms associated with plant tissues, to evaluate the long-term ecological and toxicological impacts, and to use omics-guided strategies to improve endophyte–nanoparticle synergy [51,52,53]. These are the challenges that need the crucial research at the frontier in the fight against biofilm-associated antimicrobial resistance. The main issue usually concerns metal-oxide and metallic nanoparticles used in endophyte-mediated synthesis, especially zinc oxide, copper oxide, iron oxide, and selenium, gold, and silver nanoparticles, because these are the most common biofilm inhibitors and biological activities reported in the literature [54]. Their problems include uncertain interaction with host tissues, dose-dependent cytotoxicity, organ accumulation, immune activation, oxidative stress, and poorly understood long-term ecological persistence after release into soil or water [55].

## 3. Biogenic Synthesis and Characterization of Metallic Nanoparticles from Endophytic Bacteria and Fungi

Plant-based and microbial-based synthesis serves as natural reducing as well as stabilizing agents, yielding eco-friendly metallic nanoparticles without the use of hazardous conventional methods [56]. Researchers have extensively explored the novel physical and chemical properties of nanoparticles over recent decades, sparking interest across fields like agriculture, electronics, food processing, pharmacy, and medicine [57]. Biological synthesis inside living cells offers various approaches in efficiency and yield [58]. Endophyte biogenic synthesis of metallic nanoparticles depicts a sustainable, environmentally safer approach for metal detoxification via both extracellular and intracellular mechanisms, behaving as “nano-factories” to transform ionic metals into elemental nanoparticles [15]. Metallic nanoparticles synthesized from biological approaches are the simplest, sustainable, nontoxic, and ecofriendly methods. Metallic nanoparticles are generally synthesized through extracellular or intracellular synthesis methods. In the extracellular synthesis method, fungal cell-free filtrates containing extracellular biomolecules are used for the reduction of metal ions and facilitate the nucleation, mineralization, capping, and stabilization of the synthesized nanoparticles. In this approach, the metal salt precursor is added directly to the cell-free filtrate, resulting in the synthesis of freely dispersed nanoparticles, and no additional procedure is necessary for releasing the nanoparticles from the cells. In contrast, intracellular synthesis takes up the metal salt precursor into the microbial cells or fungal mycelia, where nanoparticles are synthesized within the cellular matrix through the action of intracellular biomolecules. Consequently, following incubation, the biomass is separated from the reaction medium and subjected to an additional cell disruption or lysis step, such as sonication or chemical treatment, to release the intracellularly synthesized nanoparticles. The released nanoparticles are subsequently recovered by repeated centrifugation and purified through successive washing with the help of distilled water or ethanol to remove cellular debris and unreacted metal salts [59]. Mostly, the extracellular pathway method is considered due to its simplicity, as it is a process that occurs in a solution formed through enzyme-mediated interaction between metal ions and reductase-rich biological components [60]. During intracellular synthesis, internalized metal ions undergo enzyme-assisted nanoparticle synthesis within the cells, often yielding smaller particles than the extracellular process. The metallic nanoparticles are generally synthesized in five phases: metal ion binding—adsorption of the metal ions onto the negatively charged microbial surfaces such as peptidoglycan, lipopolysaccharide, teichoic acids, or components of the fungal cell wall. Functional groups such as carboxyl, hydroxyl, amino, and phosphate groups act as binding sites to the metal precursor, reduction process—the microbial reductase transfer the electrons from donors NADH (Nicotinamide Adenine Dinucleotide), NADPH (Nicotinamide Adenine Dinucleotide Phosphate), or other metabolites to the metal ions and converts ions into zero-valent atoms, nucleation—to form stable nuclei, growth and capping—microbial proteins, enzymes, bind to the nanoparticle surface and help in controlling aggregation, and maturation and stabilization—biomolecular coatings from microbes can keep particles stable by providing electrostatic or steric repulsion [61,62]. Metal ion recovery and residual metal concentration in the reaction medium are also important parameters for evaluating synthesis efficiency. However, these parameters are not consistently reported in the available studies. Future studies should quantify residual metal ions and recovery percentage to better assess synthesis efficiency, scalability, cost-effectiveness, and environmental safety. Endophyte-derived proteins, enzymes, polysaccharides, and other metabolites can bind to the nanoparticle surface and form a biological capping layer [63]. This layer can help prevent aggregation and may influence interactions with bacterial membranes, EPS (Extracellular Polymeric Substances), and quorum sensing pathways. However, without quantitative characterization of surface-associated biomolecules, the observed antimicrobial or antibiofilm activity cannot be specifically attributed to the biogenic capping layer. Previous studies employed a novel bacteria-engineered method for synthesizing zinc oxide nanoparticles to overcome AMR; an endophyte, *Paenibacillus species* IHC2 bacterial strain, obtained from *Indigofera heterantha* root, was used as a source of green synthesis and a stabilizing agent [64]. The secreted biomolecules involved in nanoparticle synthesis may include enzymes, proteins, polysaccharides, and other metabolites. These biomolecules can participate in metal–ion reduction, nucleation, capping, and stabilization [65]. However, their exact composition and concentration vary between endophytic species and culture conditions and are not consistently reported. Fungal strains like *Aspergillus niger* excel in extracellular secretion of hydrolytic enzymes and peptides for biosynthesizing silver nanoparticles with sharp plasmonic peaks, leveraging their robust metabolic versatility for heavy metal tolerance. In recent years, plant-derived endophytic green-synthesized metallic nanoparticles have accelerated wound-healing processes [66]. For the synthesized zinc oxide nanoparticles, constrained by the broad distribution and dose-dependent ROS (Reactive Oxygen Species) of silver nanoparticles, reproducibility may be a major challenge because of their particle size, shape, and strain variability of the fungi. Table 1 represents the studies on metallic nanoparticle syntheses using endophytic bacterial species, and Table 2 represents the studies on metallic nanoparticle syntheses using endophytic fungal species, including their particle size, shape, characterization techniques, and biomedical applications.

The morphological and chemical properties of endophyte-mediated metallic nanoparticles have been thoroughly characterized by utilizing various techniques [54]. Thorough characterization of endophyte-derived metallic nanoparticles is vital for linking structure to function and related biological activity. Key factors including nanoparticle shape, size, architecture, ligand interactions, surface chemistry, and mechanical traits dictate their intracellular uptake in targeted cells [67]. Morphology profoundly influences immune system compatibility, while high concentrations may trigger toxicity, and lower doses or concentrations often yield beneficial effects [68]. Figure 1 is a representation of plant-associated endophytes used for metallic nanoparticle syntheses and the analytical techniques used to characterize the resulting nanoparticles. For example, the physicochemical traits of CuONPs (Copper Oxide Nanoparticles) synthesized from *Laurus nobilis* L. leaf extract are morphologically stabilized by the synthesis method [69]. Physical properties of metallic nanoparticles like UiO-66 and Zn-BiOBr heterostructures have been investigated for optical applications [70]. Collectively, contemporary research underscores how the interplay of physicochemical properties and surface chemistry drives the biological roles. Ultimately, understanding these properties is crucial for biomedical applications, as it governs dispersive performance and overall functionality [71]. Key techniques include UV–vis (UV–Visible Spectroscopy) for initial confirmation through surface plasmon resonance; XRD (X-Ray Diffraction) for crystalline structure and phase purity; TEM (Transmission Electron Microscopy) and SEM (Scanning Electron Microscopy) for size, shape, and morphology; DLS (Dynamic Light Scattering) and Zeta potential for hydrodynamic size, polydispersity, and surface charge; and FTIR (Fourier Transform Infrared Spectroscopy) is used for analyzing the vibrations of functional groups from endophytic biomolecules acting as reducing, stabilizing, and capping agents [72]. The overall physicochemical characterization of metallic nanoparticles synthesized by using endophytic microorganisms is mainly influenced by the strain identity or microbial species used, temperature, precursor concentration, pH, and incubation time in the syntheses process. The strain identity or microbial species may affect the synthesis of nanoparticles due to the diverse nature of the endophytic microorganisms secreting different types of biomolecules, enzymes, and proteins that help in reducing, stabilizing, and capping the metal precursor, leading to variable shape, size, and surface chemistry. Furthermore, the concentration of the metal precursor used must be accurate to avoid excessive aggregation; if the concentration is too high, the size of nanoparticles is large, while if the concentration is too low, the nanoparticles result in irregular morphology [24]. The ionizable state of the metal precursor and microbial biomolecules is altered by the pH variation, shifting the reduction potential and capping interaction to alkaline or acidic conditions. At the last temperature is the most important aspect for the synthesis of nanoparticles, as it directs the reaction kinetics, accelerates the reduction process, and affects particle size distribution; at high temperatures, kinetics are faster, resulting in a narrower particle size distribution. Additional factors that might influence nanoparticle formation are ionic strength, dissolved oxygen, and the synthesis conditions, which modulate the redox chemistry and capping efficiency, affecting stability (zeta potential) and long-term colloidal behavior. Dissolved oxygen, endogenous ROS (Reactive Oxygen Species), and oxidase enzymes may also influence nanoparticle formation. These oxidative processes can compete with the reduction of metal ions and may affect reaction yield, particle size distribution, capping, and nanoparticle stability [73]. Therefore, the balance between reducing and oxidative processes should be considered when optimizing endophyte-mediated nanoparticle synthesis. Differences in synthesis conditions can result in differences in nanoparticle size, morphology, surface chemistry, and biological activity. Therefore, direct comparison between studies should be made carefully because experimental conditions are not standardized. These characterization techniques provide an overview of the major endophyte-mediated metallic nanoparticles and are representative of biomedical applications [74]. Figure 2 summarizes the principal of metallic nanoparticles synthesized by endophytes, including gold (Au), copper (Cu), iron (Fe), silver (Ag), titanium dioxide (TiO_2_), and zinc oxide (ZnO) nanoparticles, together with their characteristic properties including antioxidant, antimicrobial, antibiofilm, antifungal, anticancer, anti-inflammatory, wound-healing, magnetic resonance imaging, and drug delivery applications. The figure also highlights the broad therapeutic and agricultural potential of endophyte-mediated green nanotechnology.

**Table 1:** Endophytic bacteria-mediated synthesis of metallic nanoparticles: synthesis conditions, physicochemical characterization, and biomedical applications.

Endophytic Bacterial Species/ Plant Source	Metallic Nanoparticles	Synthesis Route	Characterization Techniques	Particle Shape and Size	Biomedical Application (ZOI/Inhibition Rate)	MIC/MBC Value	Reference
*Bacillus amyloliquefaciens* isolated from *Olea europaea*	AgNPs (Silver Nanoparticles)	Extracellular synthesis	UV–visible, FTIR, Zeta potential, and SEM-EDS	Spherical and rough, 24 nm	Zone of inhibition of 22 mm at 2 mg/mL concentration against *Morganella morganii*	NR	[75]
*Bacillus siamensis* isolated from *Coriandrum sativum*	AgNPs (Silver Nanoparticles)	Extracellular synthesis	UV–visible, FTIR, XRD, SEM-EDS, and TEM	Spherical, 25–50 nm	Zone of inhibition of 17.3 mm and 16.0 mm at 20 mg/mL concentration against *Xanthomonas oryzae* pv. oryzae (Xoo) strain LND005 and *Acidovorax oryzae* (Ao)	MIC of synthesized AgNps at 10 µg/mL against *Xanthomonas oryzae* pv. oryzae (Xoo) strain LND005 and *Acidovorax oryzae* (Ao)	[76]
(Five endophytes) *Bacillus cereus, Methylophilus flavus, Rhizobium pusense, Bacillus flexus, Pseudomonas aeruginosa* isolated from *Solanum lycopersicum* L.	AgNPs (Silver Nanoparticles)	Intracellular synthesis	UV–visible, FTIR, XRD, HR-SEM, and TEM-SEAD	*Bacillus flexus:* spherical, 3–28 nm; *Bacillus cereus*, 3–26 nm, spherical and cuboid 3–39 nm; and *Pseudomonas aeruginosa*: spherical 18–39 nm	AgNPs synthesized by using *Pseudomonas aeruginosa* zone of inhibition against *Bacillus thuringiensis*: 21 ± 2 mm; *Pseudomonas putida*: 28 ± 2 mm; *Azotobacter chroococcum:* 9 ± 0.8 mm; *Rhizobium* species (CV110): 11 ± 1 mm. Furthermore, AgNPs synthesized using *Bacillus flexus* and *Bacillus cereus* showed an inhibition zone of against: *Escherichia coli:* 7 ± 0.6 mm; *Bacillus licheniformis:* 10 ± 0.8 mm	NR	[77]
*Bacillus endophyticus strain* H3 isolated from (Onion)	AgNPs (Silver Nanoparticles)	Extracellular synthesis	UV–visible, FTIR, XRD, SEM-EDS, and TEM	Spherical, 4.17–26.9 nm	AgNPs with a concentration of 40 µg/mL showed antifungal activity against rice pathogenic fungal disease *Magnaporthe oryzae* with an inhibition rate of 88% in mycelial growth	NR	[78]
*Pseudomonas poae strain* CO isolated from *Allium sativum* (garlic)	AgNPs (Silver Nanoparticles)	Extracellular synthesis	UV–visible, FTIR, XRD, SEM-EDS, and TEM	Crystalline, spherical, 19.8–44.9 nm	AgNPs with a concentration of 20 µg/mL showed antifungal activity against pathogen *Fusarium graminearum* with an inhibition rate of 80.78% in mycelial growth	NR	[79]
*Brevibacillus brevis* PI -5 isolated from *Pulicaria incisa*	CuONPs (Copper Oxide Nanoparticles)	Extracellular synthesis	UV–visible, FTIR, XRD, SEM-EDX, XPS and TEM	Crystalline spherical, 2–28 nm	CuONPs with a concentration of 300 µg/mL show antifungal activity against *Aspergillus niger* with an inhibition rate of 70.2% ± 2.3%, and for *Fusarium oxysporum* it was 64.5 ± 4.1%, while the zone of inhibition against *Alternaria alternata* was 62.9 ± 0.3%	NR	[80]
*Neobacillus niacin* AUMC–B524 isolated from *Lycium shawii* (Roem and Schult leaves)	Ag_2_ONPs (Silver Oxide Nanoparticles)	Extracellular synthesis	UV–visible, FTIR, DLS, Zeta potential, XRD, EDX, Raman analysis, and TEM	Crystalline, polydisperse, and spherical shape, 13.16 nm	NR	Ag_2_ONPs showed MIC at 1–25 µg/mL against multi drug resistant wound pathogens, *Escherichia coli* (EC-3); *Staphylococcus aureus* (SA-17); *Klebsiella pneumoniae* (KP-1); *Pseudomonas aeruginosa*	[81]

**Table 2:** Endophytic fungi-mediated synthesis of metallic nanoparticles: synthesis conditions, physicochemical characterization, and biomedical applications.

Endophytic Fungal Species/ Plant Source	Metallic Nanoparticles	Synthesis Route	Characterization Techniques	Particle Shape and Size	Biomedical Application (ZOI/Inhibition Rate)	MIC/MBC Value	Reference
*Phyllosticta owaniana* KUMBMDBT-32 isolated from *Abrus precatorius*	AgNPs (Silver Nanoparticles)	Extracellular synthesis	UV–visible, FTIR, DLS, XRD, SEM-EDAX, and HR-TEM	Spherical shape, 65.81 nm	AgNPs showed antibacterial activity against *Pseudomonas aeruginosa* with a 15.1 ± 17 mm zone of inhibition and antifungal activity against *Candida albicans* with a zone of inhibition of 12.16 ± 0.28 mm	NR	[82]
*Corynespora smithi* isolated from *Bergenia ciliata*	AgNPs (Silver Nanoparticles)	Extracellular synthesis	UV–visible, FTIR, DLS, Zeta potential, XRD, SEM-EDX, and TEM	Irregular shape, 61.95 nm	AgNPs show antibacterial activity against *Staphylococcus aureus* with an inhibition zone of 20.85 ± 1.20 mm and 15.0 ± 0.14 mm against *Bacillus cereus*, while it was 18.95 ± 0.35 mm against *Bacillus subtilis*	NR	[20]
*Fusarium solani* ATLOY -8 isolated from *Chonemorpha fragrans*	AuNPs (Gold Nanoparticles)	Extracellular synthesis	UV–visible, FTIR, DLS, Zeta potential, XRD, and SEM	Spindle shape, 40–45 nm	NR, AuNPs show anticancer activity against the HeLa cell line and MCF-7 (cancer) cell line, exhibiting IC_50_ values of 1.3 ± 0.5 µg/mL and 0.8 ± 0.5 µg/mL, respectively	NR	[83]
*Fusarium chlamydosporum* MW341592 isolated from *Eucalyptus sideroxylon*	ZnONPs and AuNPs (Zinc Oxide Nanoparticles and Gold Nanoparticles)	Extracellular synthesis	UV–visible, XRD, DLS, Zeta potential, HR-TEM, and EDX	Spherical-like shape (ZnONPs) 19.3 nm and (AuNPs) 22.1 nm	NR	ZnONPs showed MIC values of 0.98 μg/mL against *Escherichia coli* and 1.95 μg/mL against *Pseudomonas aeruginosa,* while for AuNPs it was 7.81 μg/mL against *Pseudomonas aeruginosa* and it was 3.9 μg/mL against *Escherichia coli*	[84]
*Coniothyrium chaingmaiense* KUMBMDBT-25 isolated from *Euphorbia tirucalli*	AgNPs (Silver Nanoparticles)	Extracellular synthesis	Bio-spectrophotometer FTIR, DLS, Zeta potential, XRD, SEM-EDAX, and HR-TEM	Crystalline structure, 65.81 nm	AgNPs showed antibacterial activity at a concentration of 10 mg/mL against *Pseudomonas aeruginosa* with zone of inhibition 14.06 ± 0.11 mm and the antifungal activity at a concentration of 10 mg/mL against *Aspergillus flavus* was 13.03 ± 0.05 mm	Con-AgNPs showed MIC values of 0.0625 mg/mL against *Escherichia coli*, 0.125 mg/mL against *Staphylococcus aureus*, *Salmonella typhi*, and *Klebsiella pneumoniae*, while the highest MIC of 0.25 mg/mL was recorded against *Pseudomonas aeruginosa*.	[85]
*Talaromyces purpureogenus* isolated from *Pinus densiflora*	AgNPs (Silver Nanoparticles)	Extracellular synthesis	UV–visible, FTIR, PSA, XRD, and FETEM-EDS	Dispersed, triangle and round shapes, 25 nm	Tp-AgNPs showed antibacterial activity at a concentration from 25 to 100 µg/mL against *S. aureus* with a zone of inhibition 6 to 9 mm; for *B. cereus*, 8 to 11 mm; for *S. enterica* 8 to 11 mm; for *P. aeruginosa* 7.5 to 13 mm; and for *E. coli*, 8 to 11 mm. and also showed antiproliferative activity against A549 lung carcinoma cells	AgNPs showed antibacterial activity against Gram-positive bacteria with MIC of 16.12 μg/mL and against Gram-negative bacteria with MIC of 13.98 μg/mL	[86]
*Aspergillus niger* isolated from *Acalypha hispida*	ZnONPs (Zinc Oxide Nanoparticles)	Extracellular synthesis	UV spectrum, FTIR, DLS, Zeta potential, XRD, and SEM	Spherical and hexagonal shape, 31.37 ± 4.38 nm	NR	ZnONPs showed antibacterial activity against *Staphylococcus aureus* with MIC of 8–128 µg/mL	[87]

Note: NR (Not Reported); MIC (Minimum Inhibitory Concentration); ZOI (Zone of Inhibition).

The studies were incorporated based on their relevance to endophyte-mediated metallic nanoparticle synthesis and reported antimicrobial or biomedical activity. Studies were included when sufficient experimental information was available for comparison.

## 4. Mechanism of Action of Synthesized Metallic Nanoparticles Against Pathogenic Microorganism Survival, Forming Biofilm, Quorum Sensing, and Virulence Factors

Bacterial biofilms represent sophisticated survival strategies across diverse environments, from marine and terrestrial ecosystems to industrial sites. These pathogenic biofilms form multicellular communities, encapsulated in an EPS (Extracellular Polymeric Substance), protecting cells from stressors like antibiotics and oxidative damage [88]. Biofilm formation is a multistep process: the transition from free-floating planktonic cells to surface-attached aggregates involved in initial adhesion through weak interactions such as electrostatic attraction, hydrophobic interactions and van der Waals forces, followed by formation of microcolony, in which attached cells multiply and facilitate the attachment of additional bacteria, leading to large production of EPS (Extracellular Polymeric Substances) components like extracellular DNA and proteins, often co-operating host materials [89]. Mature biofilms are more resistant to drug penetration and more tolerant to antimicrobial treatment than the initial planktonic cells. The final step includes the disruption of the biofilm, in which cells detach from the biofilm and return to the planktonic state, enabling colonization of new niches and spreading persistent infection [90,91]. Pathogens such as *Salmonella typhi,* responsible for typhoid fever, form intricate biofilms that amplify infectivity [92]. *Staphylococcus aureus* creates small-colony variants within biofilms as primary drug-resistance pathogens, while Gram-negative *Klebsiella pneumoniae* form multidrug resistance biofilms [93,94]. Quorum sensing, mediated by autoinducer signaling molecules, orchestrates biofilm development and synchronized gene expression on a population scale [95]. This process extends to fungi like *Candida albicans* [96]. Even probiotics like *Bifidobacterium* employ quorum sensing to modulate metabolite secretion, including biofilms and extracellular vesicles, which can suppress virulence genes [97]. Future investigations should examine combination-based therapies, targeted biofilm disruption, and improved delivery platforms for biofilm-associated infections and industrial applications [98]. The capability of metallic nanoparticles to attach to and penetrate bacterial biofilms generally depends upon the physicochemical characteristics, as these biofilms behave as a heterogeneous and defensive barrier. Amongst the physicochemical properties, the particle size of the metallic nanoparticles is the most important aspect; smaller size penetrates more efficiently through the pores and water channels of the extracellular polymeric substances, whereas the larger size of metallic nanoparticles is retained by the outer tough layer of biofilm [99]. Metallic nanoparticle morphology also plays a role in biofilm disruption by altering the available surface area, providing greater chances of interaction [100]. The third key parameter that influences the penetration of metallic nanoparticles in biofilm is the surface charge, which plays a significant role, where positively charged nanoparticles modified through the amine groups bind to DNA and membrane components through electrostatic interactions with the negatively charged components of the extracellular polymeric substance. Overall, the most effective antibiofilm metallic nanoparticles tend to balance small size, stable dispersion, appropriate morphology, and a surface chemistry that favors strong but not irreversible interactions with the matrix, allowing them to attach, diffuse, and disrupt the biofilm more efficiently. This present review highlights the potential of metallic nanoparticles as an antibiofilm agent and emphasizes their mechanisms of action, including inhibition of quorum sensing, ROS (Reactive Oxygen Species), and degradation of biofilm matrix [101]. ROS-mediated damage and quorum sensing inhibition may act together during biofilm inhibition. Oxidative stress and membrane damage can interfere with bacterial signaling, while quorum sensing inhibition can reduce extracellular polymeric substance production and biofilm development [102]. Therefore, these mechanisms may contribute synergistically to the overall antibiofilm activity of metallic nanoparticles. Through synergistic and multi-mode of action, these metallic nanoparticles compromise both the structural stability and the biological resilience of microbes, which makes them highly effective alternatives to conventional antimicrobial agents [90]. Initially, this mechanism generally starts with the attachment of nanoparticles to the surface of the biofilm, where their physicochemical properties, including the nanoscale dimensions, surface charge, and the surface area-to-volume ratio, facilitate effective adsorption on EPS that constitutes the biofilm protective matrix. This EPS (Extracellular Polymeric Substance) matrix, composed primarily of polysaccharides, lipids, proteins, and extracellular DNA, acts as a defensive barrier against antibiotics. However, metallic nanoparticles such as copper oxide, silver, and zinc oxide exhibit strong electrostatic interactions with negatively charged biofilm components, allowing them to anchor firmly onto the surface and initiate disruption [39,103,104]. Following attachment, nanoparticles begin to penetrate the biofilm layers, a step facilitated by their nanoscale dimensions, which allow them to diffuse through water channels and pores within the biofilm architecture [105].

After penetrating the biofilm, one of the critical mechanisms of disruption involves the production of ROS, which include hydroxyl radicals, superoxide anions, and hydrogen peroxide [106]. These metallic nanoparticles, especially zinc oxide nanoparticles, under light or in dark conditions, can catalyze redox reactions that can produce these highly reactive molecules. ROS play a central role in biofilm eradication by inducing oxidative stress within microbial cells, damaging essential biomolecules such as lipids, proteins, and nucleic acids. Lipid peroxidation compromises the integrity of bacterial cell membranes, leading to increased permeability, leakage of intracellular contents, and eventual cell lysis [107,108]. Simultaneously, oxidative damage to proteins results in enzyme inactivation and metabolic dysfunction, while DNA damage interferes with replication and transcription processes, which leads to cell death [109]. Although several mechanisms have been proposed for metallic nanoparticles, the contribution of each mechanism may differ according to nanoparticle composition, size, surface chemistry, concentration, and target microorganism. Therefore, mechanisms demonstrated specifically for endophyte-mediated nanoparticles should be distinguished from mechanisms proposed from the broader metallic nanoparticle literature.

In addition to ROS-mediated damage, metallic nanoparticles exert direct interactions with microbial cell membranes. Upon contact, nanoparticles can bind to membrane proteins and phospholipids, disrupting membrane integrity through physical and chemical means [110,111]. This interaction often results in the formation of pits or pores on the cell surface, causing membrane destabilization and increased permeability. The disruption of membrane potential and ion gradients interferes with essential cellular processes such as respiration and nutrient transport. Furthermore, nanoparticles can penetrate the cytoplasm, where they interact with intracellular components, further enhancing their antimicrobial efficacy [112]. The release of metal ions, such as Zn^2+^ or Ag^+^, from nanoparticles adds another layer of toxicity, as these ions can bind to thiol groups in proteins, inhibit enzyme activity, and interfere with vital metabolic pathways, thereby amplifying the overall antimicrobial effect [113]. The mechanism of biofilm eradication and antimicrobial action of endophyte-mediated metallic nanoparticles against multidrug-resistant pathogens is demonstrated in Figure 3, being known as glitter, act as powerful weapons against multidrug-resistant pathogens [114]. AuNPs (Gold Nanoparticles) stealthily penetrate biofilms, sabotage metabolic pathways, rupture membranes, and halt EPS production, eradicating *Pseudomonas aeruginosa*, *Escherichia coli*, and *Pseudomonas putida*, stating that nanoparticles initially penetrate the extracellular polymeric substance (EPS) matrix and release metal ions that destabilize biofilm formation. Simultaneously, they generate ROS, interfere with quorum sensing pathways, disrupt bacterial membrane integrity, increase membrane permeability, and induce oxidative damage to proteins and DNA. These combined effects reduce bacterial adhesion, suppress virulence factor expression, degrade extracellular DNA and EPS components, and ultimately lead to the complete collapse and eradication of the mature biofilm. The simultaneous targeting of multiple cellular pathways minimizes the likelihood of resistance development, highlighting the therapeutic potential of endophyte-mediated metallic nanoparticles against biofilm-associated infections. Formation often correlates with cell-to-cell signaling via quorum sensing, especially under harsh conditions as seen in *Streptococcus pyogenes* [115,116]. Other than free-living microorganisms, endophytes produce bioactive metabolites and enzymes that reduce metal ions and form a natural capping layer. The endophytes possess metal tolerance mechanisms that enable efficient metallic nanoparticle syntheses under high metal ion concentrations. Combining the two properties of the endophytes and metallic nanoparticles can enhance biofilm penetration more efficiently [99,117].

Gold nanoparticles from *Aeromonas hydrophila* [118]. ZnONPs (Zinc Oxide Nanoparticles) dominate via Zn^2+^ release and ferocious ROS (O_2_^−^/H_2_O_2_/-OH) that pulverize Gram positive/negative growth and nascent biofilms [119]. Recent studies highlight endophytic bacteria and fungi as efficient biological factories for green-synthesized MNPs (Metallic Nanoparticles), particularly AgNPs and metal oxides with strong antibiofilm effects against key clinical pathogens [120]. Table 3 shows some of the metallic nanoparticles that have been discovered from plant endophytes inhibiting the biofilm formation of pathogenic bacteria. Despite these conventional methods, recent research has shifted towards nanozymes, stimulus-responsive strategies, photothermal activation design, and efficient synthesis of nanoparticles with additional functionalities targeting mature biofilms. Nanoenzymes can enhance penetration into dense biofilms while generating reactive oxygen species (ROS) through catalytic reactions [121]. The second one is stimulated activation, in which nanoparticles are generally enhanced by external or environmental stimuli such as pH, ROS, light, and bacterial enzymes [122]. A further approach is photothermal activation that combines externally activated heating with complementary mechanisms such as metal–ion-mediated metabolic disruption and nanozyme-like activity [123]. These approaches may enhance ROS production or increase nanoparticle activity under specific conditions. However, evidence specifically for endophyte-mediated nanoparticles remains limited, making these important areas for future research.

Overall, the studies summarized in Table 3 demonstrate that endophytic bacteria-mediated metallic nanoparticles, including AgNPs and TiO_2_ NPs, possess strong antibiofilm and anti-quorum sensing activities against a broad range of clinically and agriculturally important pathogens. These nanoparticles were effective against *Staphylococcus aureus*, *Escherichia coli*, *Pseudomonas aeruginosa*, *Chromobacterium violaceum*, oral pathogens, and phytopathogenic bacteria, with biofilm inhibition efficiencies ranging from approximately 20 μg/mL-dependent inhibition to over 90% biofilm removal, depending on the nanoparticle type, concentration, and target organism. Silver nanoparticles exhibited particularly strong antibiofilm and quorum sensing inhibitory activities, whereas TiO_2_ nanoparticles also demonstrated substantial biofilm suppression across multiple bacterial species. Collectively, these findings highlight the potential of endophytic bacterial-mediated metallic nanoparticles as environmentally sustainable alternatives for controlling biofilm-associated infections and bacterial virulence. Nevertheless, further studies are required to standardize synthesis protocols, elucidate molecular mechanisms, and validate their efficacy and safety under in vivo conditions to facilitate clinical and agricultural applications. The available studies provide limited quantitative evidence of biofilm eradication, as activity was reported mainly as percentage inhibition or clearance rather than standardized MBEC (Minimum Biofilm Eradication Concentration) values. Therefore, these findings were considered supportive biofilm inhibition/clearance evidence rather than quantitative MBEC data.

In Table 4, the number of available studies is limited, and the experimental designs, nanoparticle compositions, concentrations, target organisms, and endpoints differ substantially. The reported findings therefore provide evidence of antibiofilm potential in specific experimental systems rather than sufficient evidence for a generalized conclusion across endophytic fungal nanoparticles. Quantitative outcomes were reported in some studies, including concentration-dependent biofilm inhibition or clearance; however, standardized MBEC (Minimum Biofilm Eradication Concentration) measurements and directly comparable experimental designs remain uncommon. Accordingly, these findings should be regarded as representative examples that identify promising trends and research opportunities rather than as evidence of universal efficacy. Larger, standardized studies using comparable nanoparticle characterization and quantitative biofilm endpoints are required before robust cross-study conclusions can be established. However, further studies are required to standardize synthesis procedures, assess long term biosafety, and evaluate in vivo efficacy before their successful clinical translation [124].

**Table 3:** Endophytic bacteria-mediated metallic nanoparticle syntheses with their antibiofilm potential against pathogenic bacteria.

Endophytic Bacterial Species	Metallic Nanoparticles	Particle Shape and Size	Characterization Techniques	Pathogens Targeted Forming Biofilm	Biofilm Inhibition Efficacy	Reference
*Streptomyces* sp. KE4D, and *Bacillus safensis* KE4K	AgNPs (Silver Nanoparticles)	*Streptomyces* *sp* KE4D: Spherical; *Bacillus safensis* KE4K: Triangular and rod shape, 5–55 nm	UV–visible, FTIR, Zeta potential, and HR-TEM	*Chromobacterium violaceum* ATCC 12472	100–200 µg/mL AgNPs exhibiting violacein inhibition by ≥50% and <40% growth inhibition of the pathogen	[125]
*Bacillus niacini*	ZrONPs (Zirconium Oxide Nanoparticles)	Crystalline structure, 15.52 nm	UV–visible, FTIR, XRD, and SEM-EDAX	*Staphylococcus aureus*, *Escherichia coli*, *Streptococcus mutant*, *Proteus vulgaris*, *Klebsiella aerogenes*	40 µg/mL ZrONPs exhibited removal of Biofilm forming pathogen by 92.5% of *Staphylococcus aureus*, 91.5% of *Escherichia coli*, 90.5% of *Streptococcus mutant*, 83.25% of *Proteus vulgaris* and 71% of *K. aerogens*	[126]
*Streptomyces zaomyceticus* MNDV (SZ)	TiO_2_NPs (Titanium Dioxide Nanoparticles)	Spherical and polydisperse shape, 65.17 ± 3.45 nm	UV–visible, FTIR, XRD, and SEM-EDX.	*Pseudomonas aeruginosa*, *Staphylococcus aureus*, and *Escherichia coli*	Biofilm inhibition against *Pseudomonas aeruginosa* 65–85%*, Staphylococcus aureus* 70–90%, and *Escherichia coli* 60–80%	[127]
*Bacillus siamensis strain* C1	AgNPs (Silver Nanoparticles)	Not specified, ~25–50 nm	UV–visible, FTIR, XRD, SEM-EDS, and TEM	*Xanthomonas oryzae* pv. *oryzae* (Xoo) strain LND0005 and *Acidovorax oryzae* (Ao) strain RS-1	20 μg/mL is the concentration that inhibits biofilm formation	[76]
*Bacillus subtilis CG1*	AgNPs (Silver Nanoparticles)	Spherical, 72 nm	UV–visible, DLS FTIR, XRD, SEM, EDX and TEM	*P. aeruginosa and S. epidermidis*	*S. epidermidis* showed the highest eradication (~83% at MIC and ~79% at ½ MIC), whereas *P. aeruginosa* showed lower eradication, declining from ~40% at MIC to ~29% at ⅛ MIC	[128]

**Table 4:** Endophytic fungi-mediated metallic nanoparticle syntheses with their antibiofilm potential against pathogenic bacteria.

Endophytic Fungal Species	Metallic Nanoparticle	Particle Shape and Size	Characterization Techniques	Pathogens Targeted Forming Biofilm	Biofilm Inhibition Efficacy	Reference
*Aspergillus parasiticus*	AgNPs (Silver Nanoparticles)	Spherical and oblate shape, 27.7 nm	UV–visible, FTIR, Zeta potential, XRD, SEM, EDX and TEM	*Staphylococcus aureus*	50 µg/mL and 100 µg/mL AgNP showed 80% clearance rate of biofilm preventing the development of multidrug resistance in *Staphylococcus aureus*	[129]
*Botryosphaeria rhodina*	AgNPs (Silver Nanoparticles)	Spherical, <20 nm	TEM	*E. coli* and *ESBL* (Extended Spectrum β-Lactamase) *producing* strain of *E. coli*	70% inhibition in *E. coli* ATCC (25922) and 60% inhibition in ESBL-producing strains of *E. coli* at 2.5 µg/mL	[130]
*Clonostachys rosea* ZMS36	Ag-CuO NPs (Silver–Copper Oxide Nanoparticles)	Spherical, 36.63 nm	UV–visible, FTIR XRD, SEM-EDS, and TEM	MRSA (Methicillin-resistant *Staphylococcus aureus)*	Ag–CuO nanoparticles reduced MRSA biofilm formation by 33.78% at the MIC and by 18.61% at one-quarter of the MIC	[131]
*Paraconiothyrium brasiliense*	TiO_2_ NPs (Titanium dioxide Nanoparticles)	Spherical and oval, 57.39 ± 13.65 nm	FTIR, Zeta potential, XRD, FE-SEM, and TEM	*E. coli*	Combined Pb–TiO_2_ NPs + TCH treatment showed the highest biofilm inhibition (49.2 ± 3.1%) compared with TCH alone (16.34 ± 1.8%) and TiO_2_ NPs alone	[132]
*Fusarium solani*	ss–AgNPs (Small-sized Silver Nanoparticles)	Spherical, 6.13 nm	UV–visible, FTIR, Zeta potential, XRD, SEM-EDS, and TEM	*B. subtilis*, *E. faecalis*, and *S. aureus*.	ss-AgNPs showed substantial antibiofilm activity against all tested pathogens, with maximum inhibition at 1000 µg/mL: 69.14 ± 0.19% for *B. subtilis*, 73.53 ± 0.10% for *E. faecalis*, and 61.17 ± 0.88% for *S. aureus*	[133]
*Penicillium citrinum*	ZnO NPs (Zinc Oxide Nanoparticles)	Spherical, 58 nm	UV–visible, FTIR, Zeta potential, XRD, and TEM	*Staphylococcus aureus*	ZnO NPs significantly (*p* < 0.05) inhibited *S. aureus* biofilm formation, with maximum inhibition of 68.7% at 1000 µg/mL and moderate inhibition of 31.9% at 800 µg/mL. Lower concentrations showed minimal inhibition 1.89%	[134]

## 5. Descriptive and Comparative Analysis Between Endophytic-Based Metallic Nanoparticles and Plant-Based Metallic Nanoparticles

Endophyte-based MNPs (Metallic Nanoparticles) are increasingly recognized as more controllable and metabolite-rich alternatives to classical plant-extract-mediated MNPs, and recent studies from the last five years highlight clear descriptive and comparative trends between these two “green” platforms. Endophytic microorganisms such as bacteria and fungi act as an intracellular bio factory, which secretes different proteins, enzymes, secondary metabolites, and polysaccharides, which work as capping as well as reducing agent, which can support metallic nanoparticle syntheses and stability under optimized conditions [135]. In contrast, plant-based MNPs rely on crude extracts whose phytochemical composition fluctuates with species, tissue, season, and extraction protocol, often leading to slower, less reproducible synthesis and greater batch-to-batch variability in size and morphology [136]. Endophytic systems can support both intracellular and extracellular synthesis, and their long-term subculturing makes them attractive “bio factories” that decouple nanoparticle production from seasonal plant availability, whereas plant-based synthesis is operationally simpler (no aseptic microbial handling), faster to scale at low–medium sophistication, and often yields strong bifunctional coatings that enhance antioxidant, antimicrobial, or anticancer activities due to the rich phytochemical [137]. Toxicologically, both approaches generally produce less hazardous nanoparticles than purely chemical routes, but endophyte-derived MNPs may offer added advantages in metal biotransformation and stabilization, while plant-derived MNPs are more widely studied, with a larger body of in vitro and in vivo data supporting their biomedical and agricultural applications. Table 5 shows the comparative analysis of plants and endophytic microorganism-mediated metallic nanoparticles. Although the findings summarized in the table are derived from different studies varying in nanoparticle synthesis, metabolites, nanoparticle stability, antioxidant activity, antimicrobial activity, and toxicity are not universally consistent, it may vary due to the target microorganism, physicochemical properties of nanoparticles, and experimental conditions.

The comparisons presented here should be interpreted as descriptive cross-study observations rather than evidence of intrinsic superiority of one synthesis route. Differences in biological source, nanoparticle preparation, concentration, target organism, and assay methodology can substantially influence the reported activity. Therefore, direct conclusions regarding the superiority of bacterial-, fungal-, or plant-mediated nanoparticles cannot be established in the absence of standardized comparative experiments.

## 6. Commercial Prospects and Future Applications of Synthesized Nanoparticles from Endophytes in Biofilm Inhibition

Commercial and future applications of endophyte-mediated metallic nanoparticles for biofilm inhibition are beginning to move from laboratory research towards commercialization, but they are tightly linked to advances in smart design, interface control, translation frameworks, and safety evaluation [141]. Enzymes, proteins, polysaccharides, and other metabolites produced by endophytes play important roles in nanoparticle synthesis by facilitating metal–ion reduction, nucleation, surface capping, and stabilization. The composition and abundance of these biomolecules differ among endophytic species and culture conditions, and a clearer understanding of these variations remains an important area for future research; refer to Table S1. Endophyte-mediated nanoparticles are increasingly recognized by the connection of biomedical, agricultural, and environmental technologies, and recent studies suggest that their antibiofilm properties could underpin several commercial and future applications [15,33,151]. In agriculture, silver nanoparticles synthesized from endophytic bacteria such as *Bacillus siamensis* have been shown not only to inhibit biofilm formation and the motility of rice pathogens like *Xanthomonas oryzae* and *Acidovorax oryzae* but also to promote plant growth, pointing toward next-generation nano-enabled crop protectants and seed treatments that combine disease control with yield enhancement [76]. Similarly, endophytic fungi-derived metallic nanoparticles from *Talaromyces* or *Penicillium* species exhibit strong antibacterial activity and biofilm suppression, suggesting their incorporation into nano-formulated biopesticides, coatings for irrigation infrastructure, or post-harvest treatments to limit spoilage biofilms along the food chain [135,152]. Recent work on endophytic bacteria-derived silver and zirconium nanoparticles has demonstrated potent antibiofilm activity against clinically relevant pathogens and highlighted their potential use in coatings for catheters, wound dressings, and other biomedical devices [104,126]. At the same time, nano–bio interface engineering has become central to future applications, with recent studies showing precise control over ligand shells and surface charge [153,154]. These nano–bio interfaces can intensely alter nanoparticle penetration into biofilms, interaction with host tissues, and immune recognition; these principles will be crucial for tailoring the natural protein polysaccharide capping layer that accompanies endophyte-mediated synthesis. Combination therapies that pair metal or metal–oxide nanoparticles with conventional antibiotics are particularly promising, and recent studies show that nano-based drug delivery systems and hybrid nano antibiotics can enhance antibiotic penetration into biofilms, overcome efflux-mediated tolerance, and reduce required antibiotic doses, a strategy that endophyte-synthesized nanoparticles are well positioned to support because their biological coronas can improve dispersion and interaction with complex biofilm matrices [75,155,156]. Even so, most nanoparticle–antibiotic combination studies remain at the preclinical stage, and clinical evidence is still limited. Future studies should evaluate pharmacokinetics, dosing, the safety and therapeutic potential of these combinations using appropriate in vivo models before clinical translation. Although most applications are still at pre-commercial or prototype stages, the convergence of strong antibiofilm efficacy with green potentially scalable bioprocesses suggests that endophyte-based nano formulations could evolve into commercial products in sectors ranging from medical device coatings and topical antimicrobial gels to antifouling paints and smart packaging; however, full translation will depend on resolving outstanding issues around regulatory approval, large-scale production and long-term safety, and in complex real-world settings [135]. These include the need for GMP-compliant production, reproducible synthesis across batches, control over physicochemical properties, and comprehensive pharmacokinetic and toxicological profiling. In addition, regulatory approval requires well-defined quality standards, validated manufacturing processes, and evidence of safety and efficacy in relevant in vivo systems. Therefore, further work is required to bridge the gap between laboratory-scale synthesis and clinically deployable nanotherapeutics [157]. Despite their promising biomedical and technological potential, most endophyte-mediated nanoparticle systems remain at the laboratory or early preclinical stage. Reported applications should therefore be regarded primarily as potential or emerging applications rather than evidence of established commercialization or clinical translation. Evidence specifically supporting large-scale production, regulatory approval, and clinical use of endophyte-mediated nanoparticles remains limited [141,158].

## 7. Challenges and Limitations

Despite their promising antimicrobial and antibiofilm potential, endophyte-mediated metallic nanoparticles face several scientific, technical, toxicological, and regulatory limitations that constrain their large-scale applications and clinical translation. The major critical challenge is that nanoparticle synthesis using endophytic bacteria and fungi is highly dependent on biological and physicochemical variables. Strain identity, host plant species, plant tissue source, culture medium, pH, temperature, incubation period, aeration, growth phase, biomass concentration, and metal precursor concentration can influence processes such as the reduction of metal ions and the stabilization of nanoparticles [74]. Even the same endophytic strain may produce different enzymes, proteins, polysaccharides, and secondary metabolites under altered nutrient or stress conditions. Therefore, the particle size, morphology, nucleation rate, surface charge, capping chemistry, colloidal stability, and biological activity may vary significantly between batches. This batch-to-batch variability remains one of the most important barriers to reproducibility, industrial scale-up, and regulatory acceptance [159].

Another major limitation is the lack of standardized synthesis and reporting protocols [160]. Many studies report nanoparticle formation based mainly on color change after the reduction process, and UV–visible spectroscopy gives the first evidence of the formation of metallic nanoparticles, but these observations are not sufficient to confirm nanoparticle quality, purity, stability, or biological relevance [161]. For reliable comparison, each study should report the endophyte identity, accession number, host plant, plant tissue, isolation method, culture conditions, filtrate or biomass concentration, metal salt concentration, pH, temperature, reaction time, purification method, yield, storage stability, and contamination screening [24]. The gap between in vitro efficacy and in vivo performance is also substantial. Most studies rely on agar diffusion assays, minimum inhibitory concentration tests, crystal violet biofilm assays, and microscopic observation [162,163,164]. Therefore, nanoparticles showing strong activity in vitro may fail under physiological conditions. Future studies should use clinically relevant biofilm models, including wound biofilms, catheter-associated biofilms, dental biofilms, lung mucus models, urinary tract biofilm models, implant-associated biofilms, and animal infection models [165].

Toxicity and safety concerns may also remain as a critical issue. Biological synthesis does not automatically guarantee biocompatibility. The toxicity of endophyte-mediated nanoparticles depends on metal type, particle size, morphology, surface charge, coating molecules, solubility, ion-release rate, dose, exposure duration, and route of administration [166,167]. These parameters may influence the cellular uptake, intracellular transportation, biodistribution, and biopersistence. Although there are benefits of the nanoparticles, additional concerns include immunogenicity, protein adsorption, and long-term exposure, which may further lead to accumulation in the lungs, spleen, and liver [168]. For these reasons, comprehensive toxicological assessment using standardized in vitro models, followed by relevant in vivo biodistribution, pharmacokinetic, immunological, and chronic toxicity studies, is essential before biomedical translation can be justified. Silver and copper nanoparticles may exhibit strong antimicrobial activity, but excessive ROS generation can damage mammalian cell membranes, proteins, mitochondria, and DNA [136]. Therefore, antimicrobial potency should not by itself be considered evidence of therapeutic safety. Cytotoxicity testing using relevant mammalian cell lines, hemolysis assays, oxidative stress markers, inflammatory cytokine profiling, genotoxicity assays, and repeated-dose toxicity studies are necessary before biomedical application. Environmental impact must also be considered, especially for agricultural, wastewater, textile, and packaging applications [169]. Nanoparticles derived from the microorganisms are known to be effective for removing environmental contaminants. Released nanoparticles may persist, aggregate, dissolve into metal ions, accumulate in aquatic or terrestrial systems, and affect non-target microorganisms, algae, plants, or beneficial microbiota. This is particularly important because the antimicrobial activity of nanoparticles could disturb microbial ecosystems or contribute to selective pressure in environmental settings [170].

There are several concerns that are related to the environmental impact, regulatory compliance, and biocompatibility that necessitate comprehensive physicochemical characterization and biological impact assessments to ensure quality. The limitation requires the development of in situ monitoring tools, robust bioprocess optimization strategies, and greater insights into the underlying biochemical-related mechanisms that enable the reproducibility, scalability, and application-ready synthesis of nanoparticles with reliable antibiofilm efficiency.

## 8. Conclusions

The present review focuses on pathogenic biofilm-forming bacteria that display serious global health concerns due to severe antimicrobial resistance. To overcome these problems, green nanotechnology utilizes endophytic microorganisms as specialized biological entities by leveraging their natural bioactive compounds as reducing as well as stabilizing agents to reduce raw metal ions towards the formation of stable metallic nanoparticles. By inducing the mechanical disruption of the cellular walls through the formation of localized ROS (Reactive Oxygen Species) and quorum sensing inhibition pathways, these biogenic nanoparticles successfully overcome the protective extracellular matrices of pathogenic colonies. But the current evidence present in this review remains predominantly supported mainly from in vitro studies. The limited availability of in vivo studies evaluating host–nanoparticle interactions represents the most important gap in the field. Therefore, future investigation must be focused on mechanistic studies incorporating metabolomics, proteomics, combination therapeutic approaches, selective biofilm disruption, and improved drug delivery technologies for biofilm-related disease. Most importantly, future research must move beyond conventional planktonic cultures and static in vitro biofilm assays toward clinical models. Overall, endophytic-mediated metallic nanoparticles must currently be regarded as promising preclinically validated treatment options for antibiofilm therapeutics. The next phase of the research must consequently focus on the large-scale production of nanoparticles without compromising their physicochemical properties for better yield, biological entities to be reproduced in batches, and whether in vivo studies are safe or not by incorporating toxicological methods through animal models. Addressing all the gaps in the research will be critical for transforming the considerable laboratory promise of endophyte-mediated nanoparticles into safe, reproducible, and clinically relevant strategies against chronic biofilm-associated infections.

## Figures and Tables

**Figure 1:**
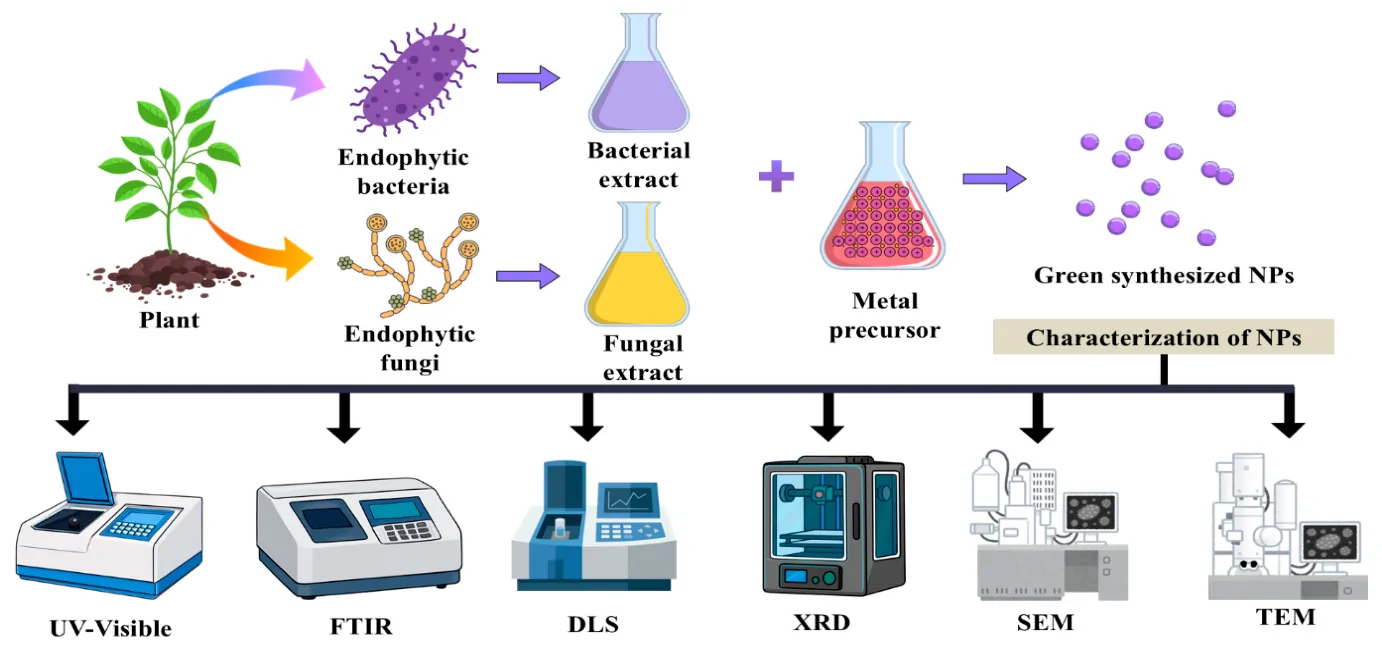
Green synthesis of metallic nanoparticles from endophytic microorganisms (fungi and bacteria), followed by physicochemical characterization.

**Figure 2:**
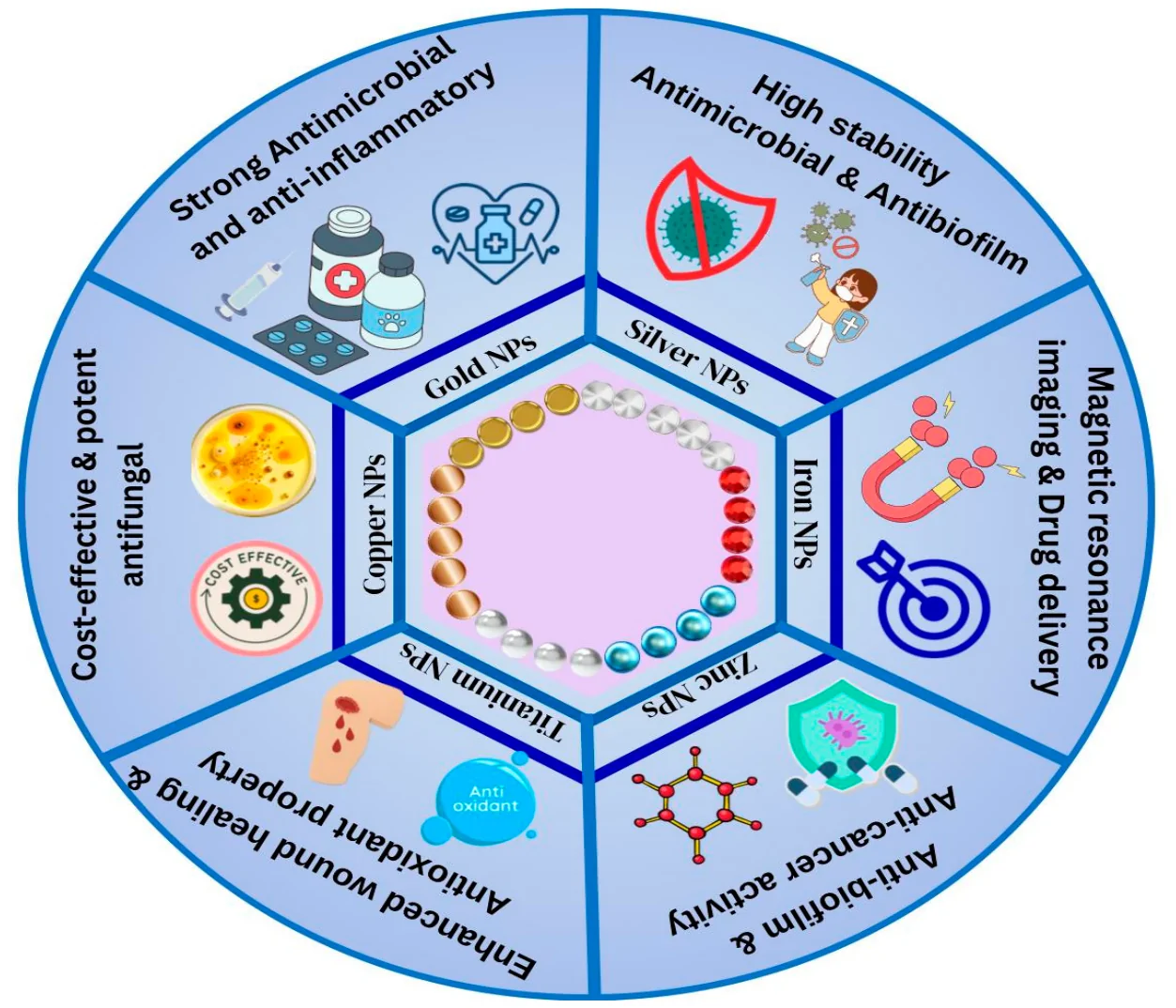
Multifunctional biomedical applications of metallic nanoparticles.

**Figure 3:**
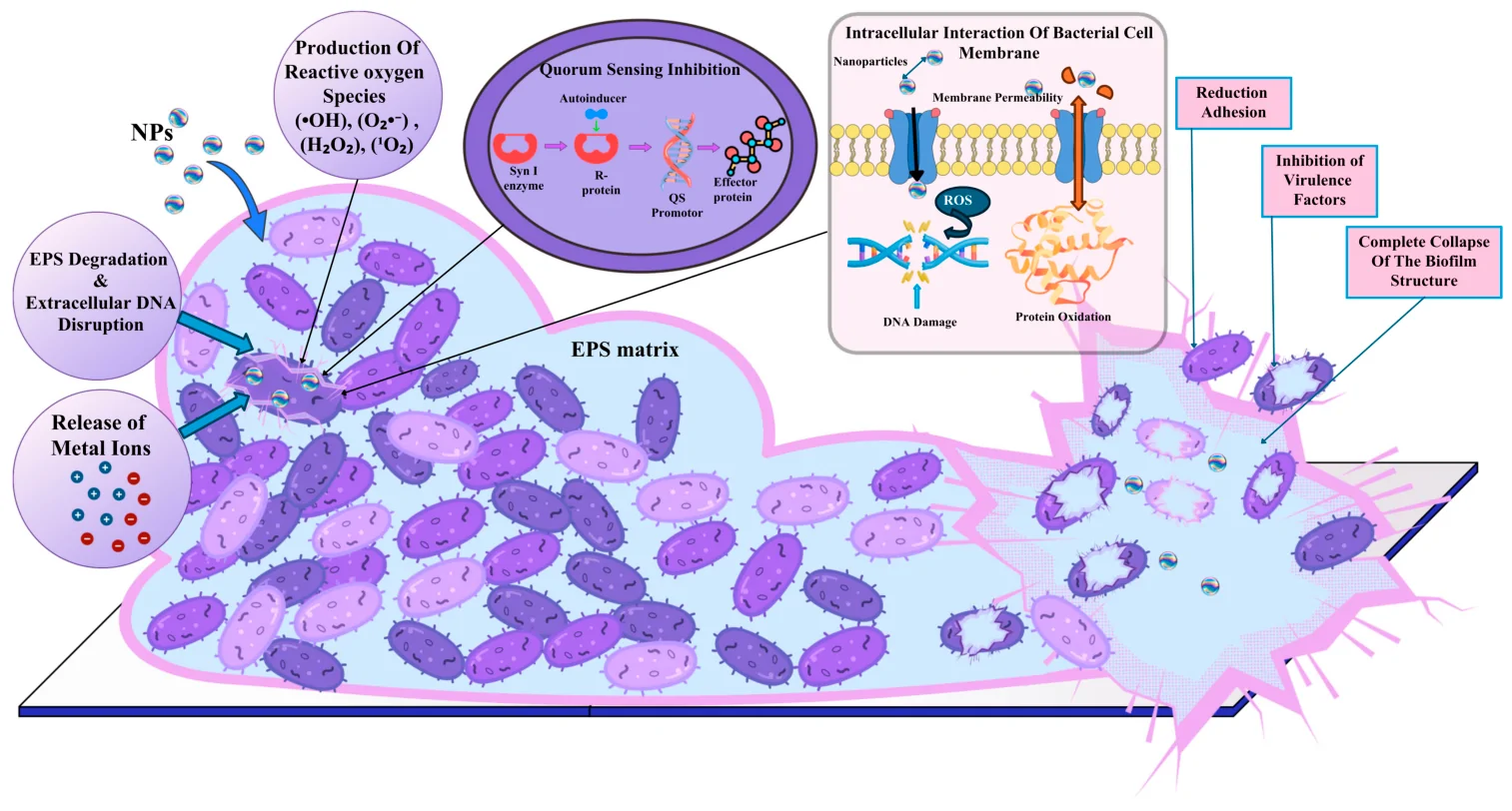
Mechanism of biofilm eradication and antimicrobial action of endophyte−mediated metallic nanoparticles against multidrug-resistant pathogens.

**Table 5:** Comparative analysis of plants and endophytic microorganisms-mediated metallic nanoparticles.

S. No	Endophytic Bacteria	Endophytic Fungi	Plant Mediated	Outcome	References
1.	Nanoparticle synthesis metabolites				
	Proteins Polysaccharides Enzymes	Fungal enzymes Proteins Terpenoids Polysaccharides phenolics	Phenolics Flavonoids Alkaloids Terpenoids Polyphenols	Act as reducing/stabilizing/capping agents Composition varies with biological source and synthesis conditions	[77,138,139,140]
2.	Nanoparticle stability				
	Enhanced by enzyme peptides and surface biomolecules for colloidal stability Long-term stability remains insufficiently investigated	Surface biomolecules may enhance stability Long-term stability remains insufficiently investigated	Phytochemicals may enhance the stability Batch-to-batch variation may occur	Stability is strongly influenced by surface chemistry and synthesis conditions Direct comparison is limited	[24,141,142,143]
3.	Antioxidant properties				
	Pontoea anthophila-mediated AgNPs DPPH: 36.99–61.31% H_2_O_2_: 28.36–56.37% ABTS: 36.99–61.31%	*Calonectria eucalypti* HECL 10-mediated AgNPs DPPH: 90% H_2_O_2_: 60.38% ABTS: 38.76%	*Martynia annua*-mediated AgNPs DPPH: 57.97–78.24% H_2_O_2_: 71.41–91.07%	Higher values were reported in selected studies Values are not directly comparable because concentrations, assays, synthesis conditions, and biological sources differed	[38,144,145]
4.	Antimicrobial properties				
	*Enterobacter hormaechei*-mediated AgNPs-MIC ranged from 1.25 to 6.00 µg/mL against the tested pathogenic and MDR microorganisms	*Aspergillus hiratsukae*-mediated AgNPs-MIC ranged from 62.5 to 1000 µg/mL against the tested bacterial and fungal strains	*Alcea rosea* leaf mediated AgNPs-MIC and MBC were 6.25 and 12.5 µg/mL, respectively, against both *S. aureus* and *S. sonnei*	Variation may reflect differences in AgNPs properties, microbial strains, and assay conditions. Values are study-specific and may vary across other reports	[146,147,148]
5.	Toxicity				
	Detailed in vivo toxicological data and long-term safety assessments are still limited	Possible immunogenic/allergenic biomolecules requires toxicological profiling	Toxicological validation remains necessary	Safety evidence remains heterogeneous Standardized in vitro, in vivo, and long-term toxicity studies are required	[24,149,150]
6.	Challenges and Limitation				
	Strain-dependent metabolite variation Batch reproducibility Limited mechanistic understanding Regulatory characterization	Mycotoxin/allergen concerns Process standardization Morphological variability Scale-up challenges	Seasonal/geographical Phytochemical variation Batch variability Process-control limitations	All platforms face reproducibility, characterization, scalability, and regulatory challenges No platform can currently be considered universally superior	[24,135,143]

## Data Availability

No new data were created or analyzed in this study. Data sharing is not applicable to this article.

## Supplementary Materials

The following supporting information can be downloaded at: https://www.mdpi.com/article/10.3390/ph19101515/s1, Table S1: Characterization of endophytic bacterial and fungal extracts and their reported chemical compounds involved in the green synthesis of metallic nanoparticles.
