# Supplementary material for: Endophyte-Mediated Green Synthesis of Metallic Nanoparticles: Mechanistic Insights, Antibiofilm Efficacy, and Translational Challenges
Source: Pharmaceuticals (Basel). 2026 Sep 23;19(10):1515. doi: 10.3390/ph19101515 (PMC13708632; doi:10.3390/ph19101515)
Supplement: Supplementary file 1 [file pharmaceuticals-19-01515-s001.zip › pharmaceuticals-4488142-supplementary.pdf]

# Supplementary file

# Endophyte-Mediated Green Synthesis of Metallic Nanoparticles: Mechanistic Insights, Antibiofilm Efficacy, and Translational Challenges

**Kaniska Singh [1], Ayush Kashyap [1], Mohammad Arham Siddiqui [1], Shabaaz Begum J. P. [1*], Prateek Gururani [2], Avinash Sharma[2], Mohammad Azam Ansari [3], Hajed Obaid A Alharbi [4] and Arshad Husain Rahmani [4*]**

1. Department of Microbiology, Graphic Era (Deemed to be University), Dehradun 248002, India
2. Department of Biotechnology, Graphic Era (Deemed to be University), Dehradun 248002, India
3. Department of Epidemic Disease Research, Institutes for Research and Medical Consultations (IRMC), Imam Abdulrahman Bin Faisal University, Dammam, 31441, Saudi Arabia
4. Department of Medical Laboratories, College of Applied Medical Sciences, Qassim University, Buraydah 51452, Saudi Arabia

* Correspondence: Shabaazbegumjp777@gmail.com, ah.rahmani@qu.edu.sa

Table S1: Characterization of endophytic bacterial and fungal extracts and their reported chemical compounds involved in the green synthesis of metallic nanoparticles.

| S. No. | Endophyte | Characterization of the endophytic extracts | Chemical compounds | Metallic Nanoparticles | Reference |
|---|---|---|---|---|---|
| | | | Endophytic Bacteria | | |
| 1. | *Bacillus amyloliquefaciens* | GC - MS | Secondary metabolites (23 compounds majorly propanedioic acid, phenyl-) | AgNPs (Silver Nanoparticles) | (Hayat et al., 2025) |
| 2. | *Clonostachys rosea ZMS36* | Not reported | Secondary metabolites (polysaccharides, proteins and organic acids) | Ag – CuONPs (Silver – Copper Oxide nanoparticles) | (Chen et al., 2025) |

| | | | | | |
|---|---|---|---|---|---|
| 3. | *Streptomyces sp* KE4D and *Bacillus safensis* KE4K | GC - MS | Cyclopropane acetic acid and 2-hexyl compounds | AgNPs (Silver Nanoparticles) | (Mohamed & Chenia, 2025) |
| 4. | *Bacillus niacini* | Not reported | Secondary metabolites (not individually identified) | $ZrO_2NPs$ (Zirconium Oxide Nanoparticles) | (Balaji & Shanmugam, 2022) |
| | | | Endophytic fungi | | |
| 1. | *Corynespora smithi* | GC - MS | Dimethylsulfoxonium formylmethylide, hexadecanoic acid | AgNPs (Silver Nanoparticles) | (Chandra et al., 2025) |
| 2. | *Aspergillus niger* | Not reported | Secondary metabolites (not individually identified) | ZnONPs (Zinc Oxide Nanoparticles) | (Abdelkader et al., 2022) |
